# Ocular exposure to blue-enriched light has an asymmetric influence on neural activity and spatial attention

**DOI:** 10.1038/srep27754

**Published:** 2016-06-13

**Authors:** Daniel P. Newman, Steven W. Lockley, Gerard M. Loughnane, Ana Carina P. Martins, Rafael Abe, Marco T. R. Zoratti, Simon P. Kelly, Megan H. O’Neill, Shantha M. W. Rajaratnam, Redmond G. O’Connell, Mark A. Bellgrove

**Affiliations:** 1 Monash Institute for Cognitive and Clinical Neurosciences (MICCN), School of Psychological Sciences, Monash University, Melbourne, Australia; 2 Division of Sleep and Circadian Disorders, Departments of Medicine and Neurology, Brigham and Women’s Hospital, Boston, USA and Division of Sleep Medicine, Harvard Medical School, Boston, USA; 3 School of Engineering, Trinity College Dublin, Dublin 2, Ireland; 4 Trinity College Institute of Neuroscience, Trinity College Dublin, Dublin 2, Ireland; 5 Faculty of Medicine, Federal University of Parana, Curitiba, Brazil; 6 Faculty of Medicine, Federal University of Mato Grosso, Cuiaba, Brazil; 7 School of Electrical and Electronic Engineering, University College Dublin, Dublin 4, Ireland; 8 School of Psychology, Trinity College Dublin, Dublin 2, Ireland

## Abstract

Brain networks subserving alertness in humans interact with those for spatial attention orienting. We employed blue-enriched light to directly manipulate alertness in healthy volunteers. We show for the first time that prior exposure to higher, relative to lower, intensities of blue-enriched light speeds response times to left, but not right, hemifield visual stimuli, via an asymmetric effect on right-hemisphere parieto-occipital α-power. Our data give rise to the tantalising possibility of light-based interventions for right hemisphere disorders of spatial attention.

The mechanisms for alertness in humans interact with those for spatial attention orienting in an intriguing fashion^1^,^2^. For example, the debilitating inattention of left space observed in patients suffering from unilateral spatial neglect subsequent to right-hemisphere damage can be temporarily overcome by phasic alerting tones^3^. Sleep deprivation in healthy participants causes relative left hemifield inattention in the visual domain^4^, while a pronounced auditory inattention to left space occurs during drowsy periods prior to sleep onset^5^. Brain imaging work in both neglect patients and neurologically healthy participants suggests that the distribution of attention between the hemifields is balanced by competitive activation between the hemispheres, specifically within a bilaterally represented dorsal network for spatial attention orienting^1^,^6^,^7^. Current models propose that this bilateral orienting network interacts with the right-hemisphere-lateralised ventral network subserving non-spatial processes such as alertness^1^,^2^ which may be preferentially innervated by the locus-coeruleus/noradrenergic (LC-NA) system^1^,^8^,^9^.

Despite demonstrations that manipulations of alertness can transiently shift spatial attention bias, neuroscience has thus far failed to identify non-invasive methods of manipulating alertness that lead to an enduring improvement in attention to left space. One promising avenue for manipulating alertness is offered by recent photobiology studies of light. Although it is recognised that light exerts powerful alerting effects on brain and behaviour, its mechanism of action has only recently been studied. Specifically, recent research has identified a set of intrinsically photosensitive retinal ganglion cells (ipRGCs) which are maximally sensitive to short wavelength (blue) light (~480 nm) and which mediate a light induced alerting signal to the human brain, in a dose dependent manner^10^,^11^,^12^. Since (a) rodent work suggests that the alerting effects of light on the brain are achieved in part via inputs from the suprachiasmatic nucleus to the LC-NA arousal system^13^,^14^, and (b) human brain imaging shows that light exposure activates key areas of right-hemisphere attention networks^15^,^16^, we asked if light-induced manipulations of alertness could be harnessed to activate right-hemisphere attention networks and thus improve the direction of attention to left space.

We hypothesized that pre-exposure to higher, relative to lower, intensities of blue-enriched light, would promote attention to the left visual hemifield, indicative of enhanced activation of right hemisphere attention networks. Neurologically healthy subjects participated in an electrophysiological study of visuospatial attention subsequent to 1 hour exposure of either low (50 lux), medium (350 lux) or high (1400 lux) intensity blue-enriched light. The effect of light on attention-related brain activity was measured via hemisphere-specific parieto-occipital α-power (8–13 Hz), a robust EEG marker of spatial attention^17^ which is also sensitive to blue light exposure^18^. Our application of multi-level statistical modelling^19^ allowed us to simultaneously model behaviour as a function of both categorical experimental conditions and pre-target neural activity, thus fully capitalizing on the richness of the single-trial EEG data.

This study yielded a number of novel and exciting findings. Light exposure enhanced response times for visual stimuli in the left, but not the right, hemifield, with a parametric speeding of left hemifield responses caused by increasing the intensity of the light. This effect did not diminish over the duration of the ~36 min attention task demonstrating that prior light exposure had an enduring impact on attention for left hemifield stimuli. This processing benefit for left hemifield stimuli was mediated by an effect of increasing light intensity on right-hemisphere parieto-occipital α-power. Our data provide the most convincing evidence yet for a direct modulatory influence of alertness on the physiological substrates of spatial attention, using a non-invasive, non-pharmacological manipulation of alertness which lasts post light exposure.

## Results

Subsequent to a 1 hour exposure to low (50 lux), medium (350 lux) or high (1400 lux) intensity blue-enriched fluorescent light (henceforth the “Light” manipulation), neurologically healthy participants performed a ~36 minute variant of the random dot motion task^20^,^21^,^22^ which involved monitoring four bilaterally distributed dot kinematograms. On each trial, after a variable delay, one of the kinematograms underwent a transition from incoherent to coherent motion (upward or downward direction) which participants were instructed to report via a speeded right-hand button press. Participants were not required to indicate either the direction or location of coherent motion. Testing occurred in the evening after ~14 hours of wakefulness. Testing an independent sample (*N* = 80) using a similar random dot paradigm between 9:30 am and 3:00 pm with no light manipulation showed that under normal daytime alertness healthy participants responded faster to coherent motion targets in the left than right hemifield [*t*(79) = −3.06, *p* = 0.003; see supplemental information for details]. The effect of prior light exposure on cortical spatial attention networks was measured via hemisphere-specific parieto-occipital α-power (8–13 Hz) recorded during the 500 ms prior to coherent motion onset^17^. We used maximum likelihood ratio tests for the fixed effects of light intensity, target hemifield and hemisphere on single trial measures of α-power and response-time (RT) (see Methods for details).

### Higher light intensity speeds target detection, specifically to left-hemifield targets

**Figure 1:**
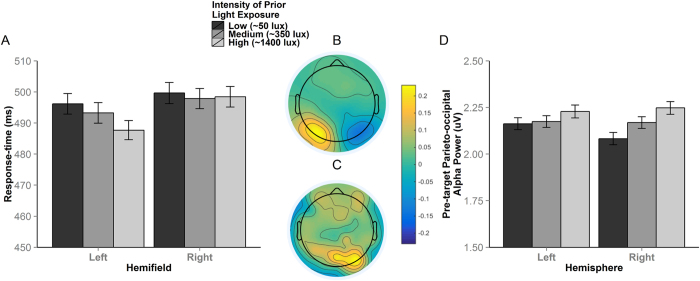
The intensity of blue-enriched light influenced response-times to targets in the left, but not right, visual hemifield (**A**). The difference in grand average post-target α-desynchronisation for left versus right-hemifield targets shows that lateral parieto-occipital electrodes were most sensitive to covert shifts in attention towards each hemifield (**B**). Increased intensity of blue-enriched light increases α-power over the right hemisphere more than left hemisphere electrodes (**C**,**D**). Note that the electrodes exhibiting strongest modulation by light in (**C**) are the same electrodes that were most sensitive to shifts in spatial attention (**B**). Error bars represent 95% CIs.

Although the main effect of Target hemifield [χ^2^(1) = 1.21, *p* = 0.272] was not significant, there was a main effect of Light [χ^2^(2) = 9.95, *p* = 0.006] on RT which was modified by a significant Light × Target hemifield interaction [χ^2^(2) = 6.81, *p* = 0.033]. Separate tests for each Target-hemifield showed no impact of Light on RTs for right-hemifield targets [all *ps* > 0.926], while left-hemifield RTs were significantly faster after high intensity Light (*M* = 490 ms, *SE* = 1.65) than after both medium (*M* = 497 ms, *ES* = 1.77) [*b* = −6.83, *SE* = 2.15, *t* = −3.17, *p* = 0.004] and low intensity (*M* = 499 ms, *SE* = 1.76) [*b* = −8.35, *SE* = 2.14, *t* = −3.90, *p* < 0.001] Light exposure (Fig. 1A). The difference between low and medium intensity was not significant [*b* = −1.53, *SE* = 2.15, *t* = −0.71, *p* = 0.758]. A lack of any significant Target-hemifield × Vertical Visual Field [χ^2^(1) = 1.11, *p* = 0.292], Light × Vertical Visual Field [χ^2^(2) = 0.82, *p* = 0.662] or Target-hemifield × Vertical Visual Field × Light [χ^2^(2) = 0.31, *p* = 0.856] interactions, indicated that the effect of Light on left-hemifield RTs was consistent regardless of whether the target appeared in the upper or lower visual field.

We next sought to determine whether the influence of the Light manipulation persisted as a function of time-on-task (trial number). The lack of any Light × Target-hemifield × Time-on-task [χ^2^(2) = 2.89, *p* = 0.236] or Light × Time-on-task interactions for left-hemifield RTs specifically [χ^2^(1) = 1.88, *p* = 0.389], indicated that the effect of Light on left-hemifield RTs persisted over the duration of the task. There was however a significant Target-hemifield × Time-on-task interaction [χ^2^(2) = 9.41, *p* = 0.002] whereby responses were slower for right than left-hemifield targets at the beginning of the task but this advantage waned with time (see Supplemental Fig. 1). This latter observation is consistent with previous reports of a rightward shift in spatial attention bias with time-on-task^4^,^23^,^24^,^25^.

### Light intensity asymmetrically modulates α-power over right-hemisphere regions that are sensitive to spatial attention orienting

Prior work has shown that night-time exposure to blue light increases α-power in waking EEG^12^,^18^,^26^. The current data support this observation since higher intensity Light increased pre-target (mean −500 ms to target onset) α-power pooled from all parieto-occipital electrodes (see Supplementary Results). Given Light had an asymmetric effect on behaviour (Fig. 1A), and since hemispheric asymmetry in posterior α-power is an established EEG correlate of the distribution of attention in space^17^, we asked whether higher intensities of Light differentially influenced α-band activity within each hemisphere. To this end we measured α-band activity over each hemisphere during the 500 ms interval immediately prior to coherent motion onsets. A scalp plot of the cumulative change in α-power across Light conditions (i.e. the sum of the change in grand average α-power between conditions [(High-Low) + (High-Medium) + (Medium-Low)]) shows that Light influenced parieto-occipital α-power over right-hemisphere electrodes more than over left electrodes (Fig. 1C). To explore this effect further, single-trial α-power measures were pooled from the four lateral parieto-occipital electrodes within each hemisphere that exhibited the strongest desynchronisation in response to covert shifts in attention towards left versus right-hemifield targets (see Experimental Methods). Light had a significant effect on α-power [χ^2^(2) = 9.80, *p* = 0.007] that was modified by a significant Light × Hemisphere interaction [χ^2^(2) = 17.0, *p* < 0.001; see Fig. 1D] while there was no main effect of Hemisphere [χ^2^(1) = 0.89, *p* = 0.891].

Follow-up contrasts showed that the Light × Hemisphere interaction was driven by a greater effect of Light on right-hemisphere than on left-hemisphere α-power (Fig. 1C,D). The effect of Light on the right-hemisphere scaled significantly in a step-wise fashion [high versus low, *b* = 0.07, *SE* = 0.007, *t* = 9.55, *p* < 0.0001; high versus medium, *b* = 0.03, *SE* = 0.007, *t* = 3.57, *p* = 0.001; medium vs low, *b* = 0.04, *SE* = 0.007, *t* = 5.97, *p* < 0.0001]. In contrast, there was no difference between medium and low Light on left-hemisphere α-power [*b* = 0.01, *SE* = 0.007, *t* = 1.66, *p* = 0.220], while the other two contrasts were significant but of smaller effect size [high versus low, *b* = 0.03, *SE* = 0.007, *t* = 4.33, *p* < 0.001; high versus medium, *b* = 0.02, *SE* = 0.007, *t* = 2.66, *p* = 0.021]. A main effect of Time-on-task [χ^2^(1) = 99.07, *p* < 0.0001] indicated α-power tended to increase over time, in line with previous findings^25^,^27^,^28^. However, there were neither Light × Time-on-task [χ^2^(2) = 1.42, *p* = 0.491] nor Light × Hemisphere × Time-on-task interactions [χ^2^(2) = 0.97, *p* = 0.615], indicating that the specific effect of Light on right hemisphere α-power persisted throughout the task.

### Higher light intensity suppresses the effect of α-power on forthcoming RTs

**Figure 2:**
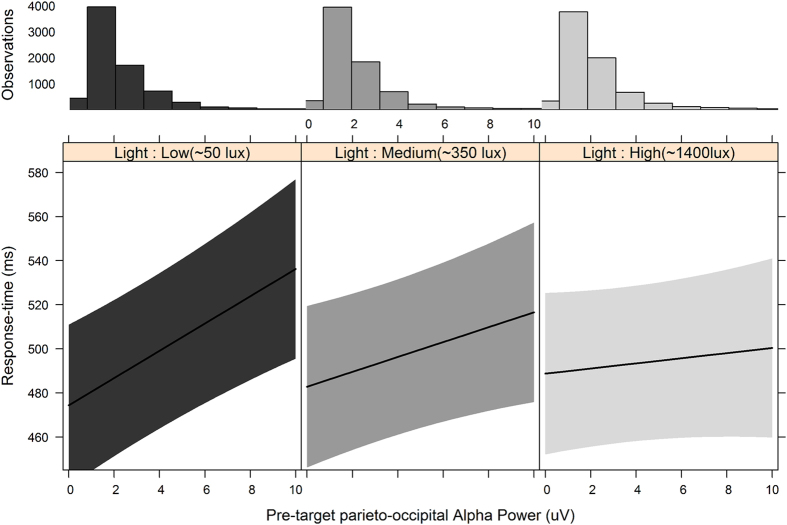
Although greater parieto-occipital α (8–13 Hz) power preceding target onset was associated with slower response times, exposure to higher intensity blue-enriched light weakened this relationship. Inserted histograms show that the distribution of observations as a function of pre-target α-power does not change across light conditions. Error bars represent 95% CIs.

It is commonly assumed that posterior α-band activity scales inversely with cortical excitability^29^,^30^,^31^,^32^,^33^. This view is supported by studies showing an inverse relationship between pre-stimulus α-power and perceptual performance, such that higher α-power is associated with slower RTs and diminished accuracy^20^,^34^,^35^,^36^,^37^,^38^,^39^,^40^. As shown above (Fig. 1D), however, exposure to higher intensity blue-enriched light actually *increased* α-power while simultaneously *improving* RTs. In a further analysis we examined the possibility that blue-enriched light exposure modifies the relationship between α-power and behavioural performance. Single-trial α-power measures were pooled from the same posterior left- and right-hemisphere electrodes used above, and entered into a model predicting RT over and above the Light × Target-hemifield effect. In line with previous studies^20^,^34^,^35^, greater α-power was generally associated with slower forthcoming RTs [χ^2^(1) = 4.89, *p* < 0.027]. Crucially, this relationship was modified by a Light intensity × α-power interaction [χ^2^(2) = 35.14, *p* < 0.0001]. Follow-up contrasts revealed that higher intensity Light suppressed the relationship between α-power and forthcoming RTs (see Fig. 2). This suppression effect scaled with light intensity [high versus medium *b* = −2.21, *SE* = 0.82, *t* = −2.70, *p* = 0.019; medium vs low *b* = −2.80, *SE* = 0.86, *t* = −3.26, *p* = 0.003; high vs low b = −5.02, *SE* = 0.85, *t* = −5.9., *p* < 0.001]. No other interactions reached significance, and there were no substantive differences in results when α-power measures were used from the left or right hemisphere separately instead of pooling across hemisphere.

### Right hemisphere α-power mediates the causal effect of light intensity on left-hemifield RTs

**Figure 3:**
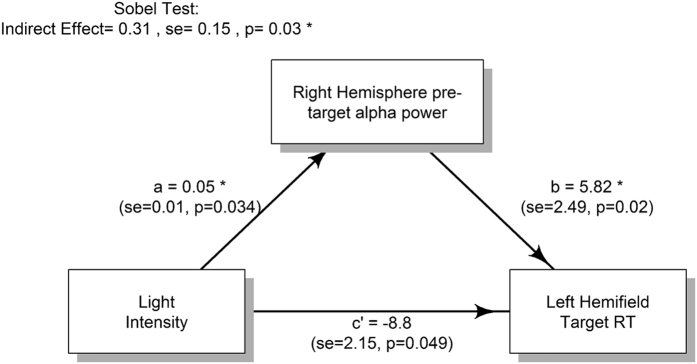
Sobel tests revealed that pre-target α-power pooled from right hemisphere parieto-occipital channels mediates the causal influence between increasing light intensity and faster RTs for left-hemifield targets. The mediation model shows that path *c’* is opposite in sign to paths *a* and *b* suggesting an ‘inconsistent mediation’ effect in line with higher Light intensity both increasing α-power (Fig. 1D) while also suppressing its slowing effect on forthcoming RTs.

We next sought to test whether the causal effect of Light intensity on left-hemifield RTs (Fig. 1A) was mediated by light’s influence on right-hemisphere α-power (Fig. 1D). Only low (~50 lux) versus high (~1400 lux) Light conditions were used as mediation analysis necessitates binary categorical predictors. Path parameters for the mediation model were calculated from the fixed effects of Light and α-power using the same linear multilevel modelling technique as above. A Sobel test^41^ demonstrated that the effect of Light intensity on left-hemifield RTs was partially mediated by parieto-occipital α-power pooled from right-hemisphere (*Indirect effect* = 0.35, *se* = 0.16, *p* = 0.03). Inspection of path parameters from the significant mediation model (Fig. 3) show that path *c’* is opposite in sign to paths *a* and *b*, indicating an ‘inconsistent mediation’ effect^42^. This accords with our observations above that higher Light intensity increases α-power (Fig. 2) while also suppressing its relationship with forthcoming RTs (Fig. 3).

In summary, we showed that greater α-power is associated with slower forthcoming RTs in line with previous studies^20^,^34^,^35^, however exposure to relatively higher intensities of blue-enriched light simultaneously increases α-power over parieto-occipital regions and improves behavioural performance for left-hemifield targets. This effect is driven in part by increasing light intensity exerting a suppressive effect on the relationship between α-power and behavioural performance, thereby facilitating faster RTs for left-hemifield targets.

## Discussion

Although it is well established that light exerts a powerful alerting influence on the human brain, its specific influence on the physiological substrates of spatial attention has not been explored. Here we show for the first time that pre-exposure to high intensity blue-enriched light can speed detection specifically for left-hemifield visual targets. This left-hemifield enhancement is driven by an enduring effect of exposure to light on right-hemisphere parieto-occipital α-power. Our results provide the most compelling evidence yet that a direct manipulation of non-spatial alertness can modulate activity within spatial attention brain networks. That these effects were achieved by manipulating the intensity of blue-enriched light, a non-pharmacological, reversible and safe stimulant, prompts consideration of future research into whether light exposure may have therapeutic benefit for disorders of spatial attention.

There are a number of novel aspects to the data reported herein. First, since previous studies of spatial attention have reported decreased α-power in the hemisphere contralateral to an attended target location, we might have expected that the light-induced enhancement of left-hemifield performance would have been coupled with a corresponding decrease in right-hemisphere α-power. Instead, however, we observed a light-induced *increase* in right-hemisphere α-power. To understand these seemingly counterintuitive findings, we tested a model by which reaction time could be predicted by the intensity of blue-enriched light, pre-target α-power and their interaction. Whereas at lower light intensity we observed the stereotypical relationship between increasing α-power and slower response times, higher light intensities weakened this effect of α-power on forthcoming response times.

So what biological process might underpin this light-induced suppression of the relationship between α-power and response times that is typically seen during tasks of spatial attention? Previous work at the intersection of circadian neuroscience and photobiology has shown that waking α-power measured at rest, which appears to be strongly modulated by the circadian system^43^, is increased during exposure to monochromatic blue light at night^12^,^18^,^26^. Lockley *et al*. hypothesized that the alerting effect of light during the night-time may be achieved by α-modulation reflecting ‘inhibition of the circadian drive for sleep’^26^. Since the participants in our study were tested in the evening after ~14 hours of wakefulness, it is possible that higher intensity blue-enriched light invoked such a mechanism, and this separate source of α-modulation merged on the scalp with the signal produced by α-generators for spatial attention. Specifically, we hypothesise that night-time exposure to higher intensities of blue-enriched light disrupts the slowing influence of α-power on forthcoming response times, by introducing greater α-modulation related to inhibition of the circadian drive for sleep. That this effect is maximal for right-hemisphere parieto-occipital α-power may explain the enhancement of attention to the left hemifield. Although previous studies of circadian and blue light modulation of resting α-power split the α-band into low and high α^12^,^18^,^26^,^44^, here the impact of blue-enriched light on spatial-attention related α-power was invariant across the 8–13 Hz range, consistent with literature on the relationship between spatial attention and α-power^17^,^39^. Nevertheless, future studies may benefit from explicitly contrasting the impact of exposure to blue-enriched light on both resting and active task scenarios as well as testing the generalisation of results reported herein to daytime light exposure.

Studies involving day-time fMRI^16^ and night-time PET^15^ have previously demonstrated that blue light disproportionately activates key right-hemisphere regions related to attention, but this is the first demonstration that these asymmetric activation patterns translate to asymmetric behavioural changes. Current models for spatial attention suggest that the distribution of attention between the hemifields is balanced by competitive activation between the hemispheres, specifically within a bilaterally represented dorsal network for spatial attention orienting^1^,^6^,^7^,^45^, that is in turn influenced by the right-hemisphere-lateralized ventral network subserving non-spatial processes including alertness^1^,^2^,^46^,^47^,^48^,^49^,^50^,^51^. This account can explain many asymmetric features of visuo-spatial attention including pseudoneglect during normal daytime alertness, reductions in responsiveness to items in left space under conditions of lower alertness, and left unilateral spatial neglect which is most common after right-hemisphere damage^1^. The non-visual effects of light on the brain are achieved in part via inputs from the suprachiasmatic nucleus to the locus-coeruleus (LC)^13^,^14^,^52^. It is therefore possible that higher intensity blue-enriched light preferentially activates right-hemisphere attention networks through the LC/noradrenergic (LC-NA) system^1^,^8^,^53^, thus leading to the asymmetric behaviour-brain effects observed herein. These observations, along with the recent findings that blue light exposure improves alertness in patients with traumatic brain injuries^54^, offer the tantalizing, though speculative, possibility that light might be harnessed as a tool to promote attention to left space in disorders involving right-hemisphere dysfunction and leftward inattention such as unilateral spatial neglect^1^,^3^ and ADHD^55^,^56^. This must be confirmed by future research.

## Methods

### Participants

Data were collected from 24 (14 female) healthy right-handed volunteers, aged 19 to 25 (M = 22.6 years), reporting normal or corrected to normal vision, no history of neurological or psychiatric disorder and no head injury resulting in loss of consciousness. Ethics was obtained from the Monash University Human Research Ethics Committee (MUHREC) prior to testing. The experimental protocol was approved by MUHREC and carried out in accordance with the approved guidelines. Informed consent was obtained from all participants prior to testing.

### Light intensity manipulation

Each participant was exposed to three different blue-enriched white light intensities (low: 50 lux; medium: 350 lux; high: 1400 lux - at eye level, vertical plane) across separate sessions in a counterbalanced order (72 testing sessions in total). Participants were seated in front of two identical light boxes (Philips EnergyLight HF3305, Philips Lighting, Eindhoven, The Netherlands) fitted with blue-enriched white lamps (17000 K, PL-L ActiViva, Philips Lighting, Eindhoven, The Netherlands)^57^. One light box was placed on a table at eye level 60 cm from the eyes. The other was positioned on the floor in front of the participant 45° degrees below eye level but pointing towards the eyes at a 110 cm distance. Light intensity (lux) was manipulated by placing the appropriate neutral density filter ‘stop’ over the source to modify the intensity (lux) of all wavelengths of light equally without altering the spectrum. Lux at eye level was verified via a lux meter (Testo 540). The only variable that differed between light conditions was the strength of the neutral density filter used.

Each participant maintained a regular sleep-wake cycle (~11:00 pm − ~7:00 am) for 7 days, with compliance confirmed both verbally and via wrist actigraphy (Actiwatch 2; Philips Respironics, Bend, Oregon, USA). Participants were instructed to avoid afternoon caffeine consumption and to maintain consistent consumption across all 7 days. On nights 5, 6 and 7 participants visited the laboratory at Monash University from 8:15 pm to 10:15 pm. Sessions comprised 10 minutes dark adaptation (complete blackout goggles), followed by 1 hour exposure to one of three light intensities (administered in a counterbalanced order). Subjective sleepiness was assessed directly before and after light exposure with the Karolinska Sleepiness Scale (KSS)^58^. Immediately following light exposure and KSS rating, participants performed the behavioural task for ~36 minutes while EEG was recorded.

Participants were not made aware of the light intensity manipulation during testing. To evaluate their perceptions of the light conditions, they completed a questionnaire directly after their third session, which asked them to judge during which of the sessions the light intensity was (a) the brightest, (b) the least bright and (c) of medium brightness. Participants then stated their confidence in these judgments, by reporting “to what extent [they] were aware each night that the light’s brightness was different to that of the other nights”, on a scale from 1 to 10 (1 - “not aware at all”; 5 - “somewhat aware, but not sure of the change”; 10 - “very aware/certain of the change”). To account for the possibility that participants were aware of the light intensity manipulation, participants’ judgments and confidence ratings were examined. Although 50% of participants correctly judged the order of light intensity exposure, there was no relationship between participants’ accuracy in these judgements and their confidence that they were correct [incorrect *mean confidence* = 6.25, *SD* = 2.93, *n* = 12; correct *mean confidence* = 7.67, *SD* = 2.5, *n* = 12. Robust *t*-test alternatives were used since confidence distributions were not normal^59^ - bootstrapped Yuen-Welch test with 20% trimmed means and windsorized variances: *t* = 1.62, *p* = 0.083; Bayesian *t*-test analogue: posterior mean confidence difference = 1.49, 95% HDI = −0.94 to 3.91]. Nor was there any effect of judgment accuracy or Light intensity on change in KSS subjective sleepiness scores after light exposure [Light intensity χ^2^(2) = 0.75, *p* = 0.688; Judgment Accuracy χ^2^(2) = 0.29, *p* = 0.590; Light intensity × Judgment Accuracy χ^2^(2) = 1.23, *p* = 0.539]. Our finding that accuracy in judging light intensity bore no relationship to subjective sleepiness ratings as a function of light intensity, together with an absence of a relationship between accuracy and confidence, suggest the absence of any expectancy effects or demand characteristics^60^.

### Behavioural paradigm

**Figure 4:**
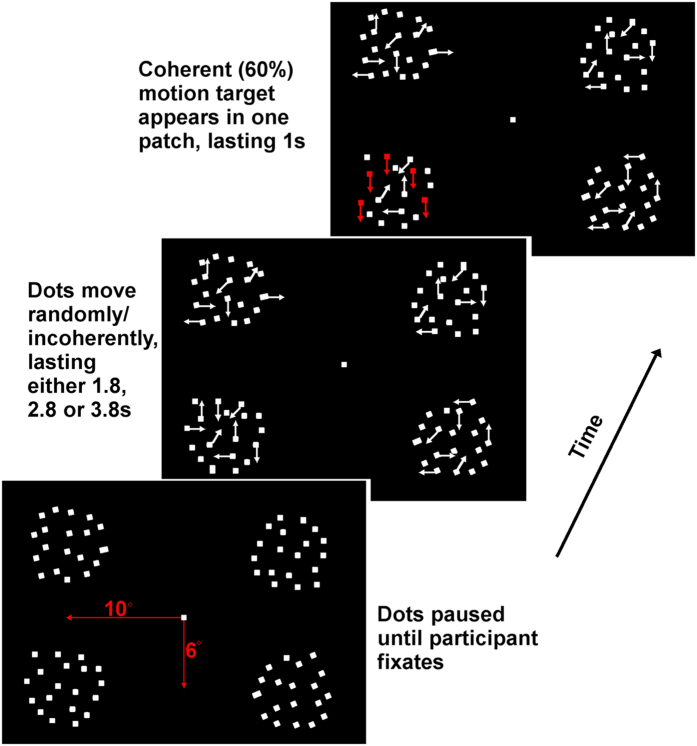
Schematic of a single trial. Participants fixated on the central dot and monitored the peripheral patches of randomly moving dots for instances of coherent motion (either upward or downward). Participants responded to motion targets via a speeded button press. Coherent motion targets only occurred in one of the four patches, once per trial. The pre-target random motion lasted either 1.8, 2.8 or 3.8 s, chosen randomly on a trial-by-trial basis.

Participants were seated in a darkened room, 56 cm from a 21 inch CRT (85 Hz, 1024 × 768 resolution) to perform a novel variant of the random dot motion (RDM) task^20^,^21^,^22^ where they fixated centrally and monitored 4 peripheral patches (one in each quadrant) of 150 moving dots for targets defined by a seamless transition from random motion to coherent motion in an upward or downward direction (see Fig. 4). Upon detecting a target, participants made a speeded button press with their right index finger. Since response hand was held constant across the three repeated-measures levels of Light, response hand cannot have influenced the key effects of Light on RT and α-power observed herein. Stimuli appeared white (RGB: 221) against a black background. The fixation mark was a central 5 × 5 pixel square. The circular dot patches were of 8 degrees diameter with the centre of each patch situated 6 degrees below or above and 10 degrees to the left or right of the central fixation point (see Fig. 4). During random motion, 150 dots per patch (each dot 6 × 6 pixels) were placed at random and independent positions within each of the patches at a rate of 21.25 frames/s. During coherent motion targets, 60% of these dots were randomly selected on each frame to be displaced by a fixed distance of 0.282 degrees in either a downward or upward direction on the following frame, resulting in a motion speed of 6 degrees/s. The four dot patches and fixation mark remained on screen throughout the entire task, however dot motion paused (i.e. all dots became stationary) between trials. When dot motion was paused, stationary dots were set slightly darker (RGB: 181) to account for the absence of the 21.25 frames/s flicker. The motivation behind using relatively high dot coherence (60%) was to ensure fast RTs with relatively low variability to enable detection of subtle processing differences between left and right hemifield targets.

Participants completed 336 trials containing no fixation breaks during each session. Each trial consisted of a period of random motion (initiated on fixation and lasting 1.8, 2.8 or 3.8 s) followed by a coherent motion target (terminated directly after a response or after 1 s). Targets only appeared once within one of the 4 patches/quadrants on any given trial. If a fixation break occurred during a trial (either a blink or a gaze deviation >4 degrees from centre, detected via EyeLink1000, SR Research Ltd), the task paused (stationary dots), and text (dark grey, RGB: 109) appeared at fixation for 200 ms reminding participants to “keep [their] eye on the spot”, before the trial restarted. The 24 possible trial types (each a combination of one of the 3 periods of random motion, 4 target locations, and 2 coherent motion directions), occurred in a pseudorandom order with the constraint that each different trial type arose twice every 48 trials. Following the coherent motion target, all dots paused and remained stationary on the screen for 100 ms before a “blink now” message (RGB: 109; 200 ms) appeared at fixation. Stationary dots then remained onscreen for a further 400 ms fixation period before the next trial began. The paradigm was run on a 32-bit windows XP machine using MATLAB (MathWorks) and the Psychophysics Toolbox extensions^61^,^62^,^63^. Paradigm scripts can be found online.

### Data processing

Data were processed using a combination of custom scripts and EEGLAB^64^ routines implemented in MATLAB (MathWorks). All processing scripts used for the current study can be found online (https://github.com/DanielPNewman/BlueEnrichedLightRepo). Continuous EEG was acquired from 65 scalp electrodes using a Brain Products BrainAmp DC system digitized at 500 Hz. A 35 Hz low-pass filter was applied offline using 4 th order Butterworth filters, noisy channels were interpolated (spherical spline) and the data were re-referenced to the average reference. Epochs were then extracted from the continuous data from −700 ms to 1200 ms around target onset, and baselined with respect to −100 to 0 ms before target onset. Power in the α-band was calculated following the methods by Thut and colleagues^17^. Each epoch was band-pass filtered to α range (8–13 Hz), rectified (converted to absolute values) and trimmed to exclude the 200 msec at the beginning and end of the epoch in order to eliminate filter warm-up artefacts. Data were then smoothed by averaging inside a moving window of 100 ms, moving forward in 50 ms increments.

### Electrode selection for pre-target α analysis as a function of hemisphere

Since short-wavelength light has been shown to modulate α-power at rest^18^ and pre-target parieto-occipital α-power is a robust marker of attentional deployment between the hemifields^17^, the current study focused on parieto-occipital α-power measured during the interval immediately prior to coherent motion onsets (mean −500 ms to target onset). The difference in grand average α-desynchronisation elicited by left- versus right-hemifield targets indicated that lateral parieto-occipital electrodes were the most sensitive to covert shifts in attention towards each hemifield (see Fig. 1B), in line with previous work^17^,^25^,^40^,^65^,^66^,^67^,^68^,^69^. Post-target α-desynchronisation was used to select individualized parieto-occipital electrodes for α-power analysis, ensuring α was measured from electrodes most sensitive to spatial orienting. Since light exposure asymmetrically influenced the RT measure of spatial attention orienting (Fig. 1A), individualized electrodes were chosen on a per-session basis. This approach focuses on the parieto-occipital regions while permitting some variation in electrode selection between sessions to account for: (a) subtle individual differences in cap fit, scalp morphology, and cortical folding between participants, and (b) subtle differences in spatial attention processing related to the light manipulation. Four electrodes per hemisphere with maximal difference in mean post-target (50–600 ms) α-desynchronisation for contralateral versus ipsilateral targets (calculated from average post-target α waveforms for each session) were selected from the 16 lateral parieto-occipital electrodes (left hemisphere P1, P3, P5, P7, PO3, PO7 PO9, O1; right-hemisphere P2, P4, P6, P8, PO4, PO8, PO10, O2).

### Inferential analysis

Inferential statistics were calculated using a combination of custom scripts and packages in R. All R scripts and data for reproduction of results can be found online (https://github.com/DanielPNewman/BlueEnrichedLightRepo). Target detection accuracy was at ceiling (mean = 97.4%). We used multilevel linear modelling and maximum likelihood ratio tests via the *lme4* package^70^ to test for fixed effects of light intensity, target-hemifield and hemisphere on single trial measures of RT and α-power data. Follow-up contrasts were performed as appropriate using the *multcomp* package^71^. This multilevel approach was preferred over classic ANOVA methods as it accommodated dependent data due to repeated measures^19^ and allowed random intercepts to account for the nesting of each cerebral hemisphere (for α-power measurements) inside participants who were nested inside different Light Condition Orders (6 possible orders administered in a counterbalanced manner). To identify the final random effect structure we first followed the criteria of Barr *et al*.^72^,^73^ by fitting a maximal model including by-subjects random slopes of each fixed effect and crossed factors of Light Intensity (low, medium, high), Incoherent Motion Period (1.8, 2.8, 3.8 seconds), Motion Direction (upward, downward), Target Location (1 of 4 quadrants) and Trial Number (1 to 336). This maximal random effects structure failed to converge, however, showing the maximal model was over-parameterized^74^,^75^. Therefore, we identified a parsimonious^74^ random effect structure using an iterative procedure^76^ which employed likelihood ratio tests to compare models without each particular random effect to the model including the random effect, and retained only the random effects which both improved the model fit and did not lead to failure to converge. This lead to the inclusion of by-subjects random slopes of Hemifield and pre-target alpha power, but the exclusion of random slopes of Light, when modelling RT, and the exclusion of random slopes for Light and Hemisphere when modelling pre-target alpha power. Fixed effect plots were created using the *Effects* and *ggplot2* packages^77^,^78^.

As described above, all completed trials were uncontaminated by eye blinks or fixation breaks. Trials were excluded from analysis if: (a) RTs were < 200 ms (pre-emptive responses) or >1000 ms (responses after coherent motion offset); (b) broad-band (0–35 Hz) EEG from any channel exceeded +/−100 μV during the interval from −500 ms to 0 ms before target onset for pre-target α analysis, or during the interval from −100 ms before target onset to 100 ms after RT for the α desynchronisation analysis which was used to select electrodes most sensitive to covert shifts in attention (see below). The distribution of single trial α-power measures had strong positive skew so they were log transformed to normality for any analysis in which α-power was the criterion/dependent variable. RT data did not require transformation. Outlying data points (greater than 3 standard deviations from the participants’ conditional mean) were removed from single trial RT and α-power measures prior to inferential analysis.

## Additional Information

**Data availability**: Analysis scripts and paradigm code (https://github.com/DanielPNewman/ BlueEnrichedLightRepo) as well as the raw data (https://dx.doi.org/10.4225/03/574CEA1FAFB69) are open access and available under a Creative Commons Attribution-NonCommercial-ShareAlike 3.0 International License.

**How to cite this article**: Newman, D. P. *et al*. Ocular exposure to blue-enriched light has an asymmetric influence on neural activity and spatial attention. *Sci. Rep.* **6**, 27754; doi: 10.1038/srep27754 (2016).

## Supplementary Material

Supplementary Information
