# Corrigendum: Ocular exposure to blue-enriched light has an asymmetric influence on neural activity and spatial attention

**DOI:** 10.1038/srep44958

**Published:** 2017-03-23

**Authors:** Daniel P. Newman, Steven W. Lockley, Gerard M. Loughnane, Ana Carina P. Martins, Rafael Abe, Marco T. R. Zoratti, Simon P. Kelly, Megan H. O’Neill, Shantha M. W. Rajaratnam, Redmond G. O’Connell, Mark A. Bellgrove

Scientific Reports 6: Article number: 2775410.1038/srep27754; published online: 06 13 2016; updated: 03 23 2017

The Acknowledgements section in this Article is incomplete.

“This study was supported by grants from the Australian Research Council (FT130101488; DP150100986). The authors thank Petra C van den Bogert for help with data collection”.

should read:

“This study was supported by grants from the Australian Research Council (FT130101488; DP150100986). The authors thank Petra C van den Bogert for help with data collection. This work was also supported by an International Research Staff Exchange Scheme (612681) of the EU 7th Framework Programme”.

