# Supplementary material for: Ocular exposure to blue-enriched light has an asymmetric influence on neural activity and spatial attention
Source: Sci Rep. 2016 Jun 13;6:27754. doi: 10.1038/srep27754 (PMC4904199; doi:10.1038/srep27754)
Supplement: Supplementary Information [file srep27754-s1.pdf]

Supplemental Results

Ocular exposure to blue-enriched light has an asymmetric influence on neural activity and spatial attention

Daniel P. Newman[1], Steven W. Lockley[1,2], Gerard M. Loughnane[3,4], Ana Carina P. Martins[1,5], Rafael Abe[1,6], Marco T. R. Zoratti[1,6], Simon P. Kelly[7], Megan H. O'Neill[1], Shantha M. W. Rajaratnam[1,2], Redmond G O'Connell[4,8,] Mark A Bellgrove[1]

[1] Monash Institute for Cognitive and Clinical Neurosciences (MICCN), School of Psychological Sciences, Monash University, Melbourne, Australia.

[2] Division of Sleep and Circadian Disorders, Departments of Medicine and Neurology, Brigham and Women's Hospital, Boston, USA and Division of Sleep Medicine, Harvard Medical School, Boston, USA.

[3] School of Engineering, Trinity College Dublin, Dublin 2, Ireland.

[4] Trinity College Institute of Neuroscience, Trinity College Dublin, Dublin 2, Ireland.

[5] Faculty of Medicine, Federal University of Parana, Curitiba, Brazil.

[6] Faculty of Medicine, Federal University of Mato Grosso, Cuiaba, Brazil.

[7] School of Electrical and Electronic Engineering, University College Dublin, Dublin 4, Ireland.

[8]School of Psychology, Trinity College Dublin, Dublin 2, Ireland.

Correspondence: Daniel. P. Newman; daniel.newman1@monash.edu

Conflicts of interest: none declared.

Additional Footnotes: Analysis scripts and paradigm code are open source, https://github.com/DanielPNewman/BlueEnrichedLightRepo. Raw data are available to reviewers only via this *private* link https://monash.figshare.com/s/7226135ab46465de3e4f and will be made public *after* this manuscript is accepted and published. The open source code/materials and data will be available under a Creative Commons Attribution-NonCommercial-ShareAlike 3.0 International License.

Supplemental Results

Hemifield by Time-on-task interaction on reaction-time (RT)

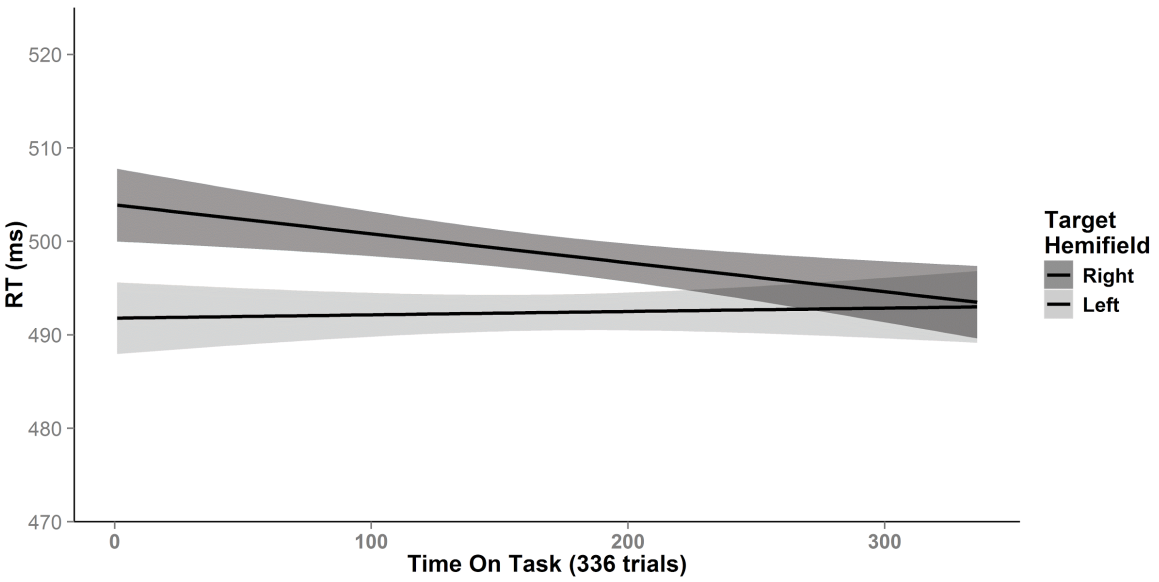

Supplemental Figure 1. A left-hemifield RT advantage was present during the beginning of the task but became smaller with time-on-task and disappeared during the second half of the session. This is consistent with the rightward shift in spatial attention bias with time-on-task that has been reported previously[1–5].

The plot above depicts data from all three Light conditions since Light did not moderate the Hemifield × time-on-task effect. Simple effects of time-on-task on RTs from each Hemifield show that the Hemifield × Time-on-task interaction was driven by an improvement in right-hemifield RTs over time [b= -0.03, SE=0.009, t= -3.28, p=0.002] while the slowing of left-hemifield RTs over time was not significant [b= -0.009, SE=0.009, t= 1.01, p=0.525]. Note: Since time-on-task may be correlated with both practice effects (RTs may become faster over time as participants' skill on the task improves) and with declining alertness (RTs may become slower over time as alertness decreases), it could be the case that an overall practice effect tends to improve RT over time, but this effect is cancelled out for left hemifield targets only due to the asymmetric behavioural effect on spatial attention of

declining alertness with time-on-task. As time-on-task is confounded with practice effects and alertness it is difficult to disentangle the degree to which each of these influences RT as function of hemifield. This is why the direct manipulation of alertness via night-time exposure to blue-enriched light as presented in the current manuscript, is an important addition to the spatial attention/alertness literature.

Significant leftward RT bias under normal daytime alertness in an independent sample of healthy participants

An independent sample (*N*=80) of healthy participants completed a random dot paradigm similar to that reported in the main manuscript, with the following differences: (a) testing occurred between 9:30am and 3:00pm under normal daytime alertness levels; (b) there was no light manipulation; (c) only two lower visual-field dot patches were used, with the same characteristics as the lower visual-field patches in the main manuscript; (d) coherence set at 60%. A repeated-measures *t*-test on the mean participant-level reaction-times for left vs. right hemifield targets showed that under normal daytime alertness healthy participants responded faster to coherent motion targets in the left (*M*=571ms , *SE*=11) than right (*M*= 586, *SE*=12) hemifield [$t(79) = -3.06$, $p = 0.003$].

The effect of Light on α-power pooled from all parieto-occipital electrodes

Prior work shows that exposure to short-wavelength (blue) light increases α-power in waking EEG at rest[6–8]. The current data support this with a significant effect of Light exposure on α-power (mean -500ms to target onset) pooled from all parieto-occipital electrodes (Pz, P1, P2, P3, P4, P5, P6, P7, P8, POz, PO3, PO4, PO7, PO8, PO9, PO10, O1, O2, Oz). Since α-power was the criterion variable here, the pooled α measures were log

transformed to a normal distribution and outliers removed leaving 22,355 observations for analysis. The main effect of Light across all parieto-occipital electrodes [$\chi^2(2) = 7.964$, $p = 0.0187$] was broken down with contrasts via glht() in the *multcomp* package[9] revealing that parieto-occipital α-power was significantly greater after high intensity light exposure than either low [$b = 0.04$, $SE = 0.006$, $t = 6.21$, $p < 0.001$] or medium intensity exposure [$b = 0.02$, $SE = 0.006$, $t = 3.90$, $p < 0.001$], and the increase in α-power between low and medium intensity light exposure was also [$b = 0.01$, $SE = 0.006$, $t = 2.35$, $p = 0.049$].

References

1. Benwell, C. S. Y., Harvey, M., Gardner, S. & Thut, G. Stimulus- and state-dependence of systematic bias in spatial attention: Additive effects of stimulus-size and time-on-task. *Cortex* **49,** 827–836 (2013).
2. Dodds, C. M. *et al.* The Effects of Time-on-Task and Concurrent Cognitive Load on Normal Visuospatial Bias. *Neuropsychology* **22,** 545–552 (2008).
3. Manly, T., Dobler, V. B., Dodds, C. M. & George, M. A. Rightward shift in spatial awareness with declining alertness. *Neuropsychologia* **43,** 1721–1728 (2005).
4. Matthias, E. *et al.* Attentional and sensory effects of lowered levels of intrinsic alertness. *Neuropsychologia* **47,** 3255–64 (2009).
5. Newman, D. P., O'Connell, R. G. & Bellgrove, M. A. Linking time-on-task, spatial bias and hemispheric activation asymmetry: A neural correlate of rightward attention drift. *Neuropsychologia* **51,** 1215–1223 (2013).
6. Lockley, S. W. *et al.* Short-wavelength sensitivity for the direct effects of light on alertness, vigilance, and the waking electroencephalogram in humans. *Sleep* **29,** 161–8 (2006).
7. Rahman, S. A. *et al.* Diurnal spectral sensitivity of the acute alerting effects of light. *Sleep* **37,** 271–81 (2014).
8. Zaidi, F. H. *et al.* Short-Wavelength Light Sensitivity of Circadian, Pupillary, and Visual Awareness in Humans Lacking an Outer Retina. *Curr. Biol.* **17,** 2122–2128 (2007).
9. Hothorn, T., Bretz, F. & Westfall, P. Simultaneous Inference in General Parametric Models. *Biometrical J.* **50,** 346–363 (2008).
